# Modified cation-exchange membrane for phosphate recovery in an electrochemically assisted adsorption–desorption process†

**DOI:** 10.1039/c9cc09563b

**Published:** 2020-03-27

**Authors:** Kostadin V. Petrov, Laura Paltrinieri, Lukasz Poltorak, Louis C. P. M. de Smet, Ernst J. R. Sudhölter

**Affiliations:** Delft University of Technology, Department of Chemical Engineering Van der Maasweg 9 2629 HZ Delft The Netherlands; Wetsus – European Centre of Excellence for Sustainable Water Technology Oostergoweg 9 Leeuwarden 8932 PG The Netherlands; Department of Inorganic and Analytical Chemistry, Faculty of Chemistry, University of Lodz Tamka 12 91-403 Lodz Poland lukasz.poltorak@chemia.uni.lodz.pl; Laboratory of Organic Chemistry, Wageningen University & Research Wageningen The Netherlands

## Abstract

A novel ion separation methodology using a cation-exchange membrane modified with iron oxide nanoparticles (Fe_3_O_4_ NPs) coated with polyhexamethylene guanidine (PHMG) is proposed. The separation is performed in an electrodialysis cell, where firstly phosphate is electro-adsorbed to the PHMG@Fe_3_O_4_ NP coating, followed by a desorption step by applying an electric current.

## Abstract 2

A novel ion separation methodology using a cation-exchange membrane modified with iron oxide nanoparticles (Fe_3_O_4_ NPs) coated with polyhexamethylene guanidine (PHMG) is proposed.

## Graphical abstract

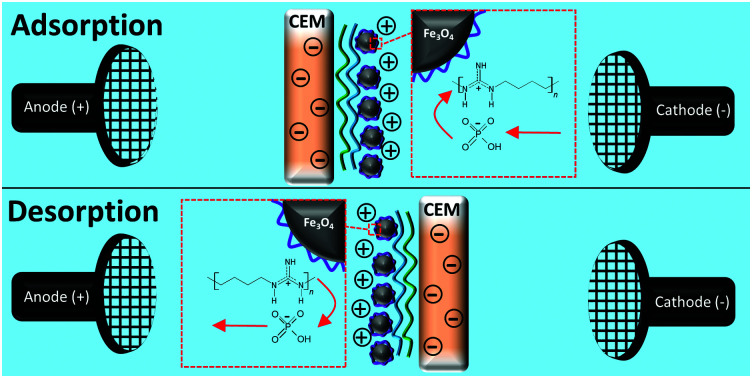

Phosphorus (P), is a fundamental element in the fertilizer industry.^1^ Over the last decades, the demand for phosphate has grown exponentially and its production has increased in response to the growing world population and higher food demand.^2^ Consequently, natural resources are getting depleted, and the predicted time for phosphate shortage ranges from 100 to 400 years.^3,4^ Therefore, the European Union has placed phosphate rock in the top 20 of the list of critical raw materials.^5^ On the other hand, eutrophication is an undesirable process caused by phosphate release to waterbodies. It causes not only algae blooms,^6,7^ but it is also considered as a threat to human health when found at elevated concentrations.^8–10^ Given the above, there is a high demand for a sustainable way of removing and recovering phosphate from wastewater. For this purpose different technologies have been developed, including biological treatments,^11^ crystallization,^12,13^ flotation,^14^ membrane,^15,16^ and adsorption-based processes.^17^ Electrochemistry, especially when combined with solar cell technology, holds a special role in water treatment technology as it allows for controlled ion removal using techniques based on electrodialysis (ED) or capacitive deionization (CDI).^18^ ED is used to transport ions from one solution (known as feed), through an ion-exchange membrane (IEM) to another solution (known as receiving phase) under the influence of an applied electric current to (pre-)concentrate the desired chemical species.^19^ Capacitive deionization (CDI) is a technology to deionize water by applying an electrical potential difference over two electrodes having a high electroactive surface area – often made out of porous carbon. In this process, the ions are removed from the water and stored in the electrical double layer of the used electrodes.^20^ The performance of ED and CDI can be further boosted by the chemical modification of the ion-exchange membranes^21^ or the electrodes,^22^ respectively. Polyelectrolytes used as a modifier play a special role in this regard.^21,23,24^ The chemical composition of these unities can be altered with the help of synthetic chemistry, whereas their placement at the surfaces follows simple protocols such as the layer-by-layer (LbL) deposition technique (governed by electrostatic interactions).

In this study, the benefits originating from an ED process and an adsorption process are combined. Adding the properties of ion-exchange membranes to high surface area adsorbents in a form of nanoparticles (NPs), and using electrochemistry as the main driving force for ion separation, a novel methodology for ion recovery is obtained. A schematic representation of the functionalized membrane can be found in Fig. 1A. A cation-exchange membrane (CEM) was used as a barrier for anions and as a support for iron oxide nanoparticles (Fe_3_O_4_ NPs) coated with polyhexamethylene guanidine (PHMG), a polycation. The coating process and characterization of the NPs are described in Section S4 of the ESI.† This polyelectrolyte was chosen because of its selective interaction with phosphate.^25^ Two other polyelectrolytes, polyethyleneimine (PEI) and poly(styrene sulfonate) (PSS) were used as intermediate layers aiming to increase the surface roughness and the charge density of the membrane. The surface interactions between polyelectrolytes are governed by electrostatic forces and can be summarized as (i) having a low p*K*_a_ value (≈1), the exposed sulfonate ion-exchange sites of a CEM are virtually always deprotonated;^26^ (ii) branched PEI was used as an intermediate layer and is positively charged at pH ≲ 11, and hence, assures a positive charge over a wide pH range;^27,28^ (iii) PSS introduces the desired negative charges (again given by the low p*K*_a_ of the sulfonate groups);^26^ (iv) Fe_3_O_4_ NPs modified with PHMG are positively charged – the p*K*_a_ of guanidinium is about 13.^23^ (v) The modification of Fe_3_O_4_ NPs was performed at pH = 9.5 being well above the iso-electric point of iron oxide^29^ and at the same time assuring a positive charge of the PHMG chain.

**Fig. 1:**
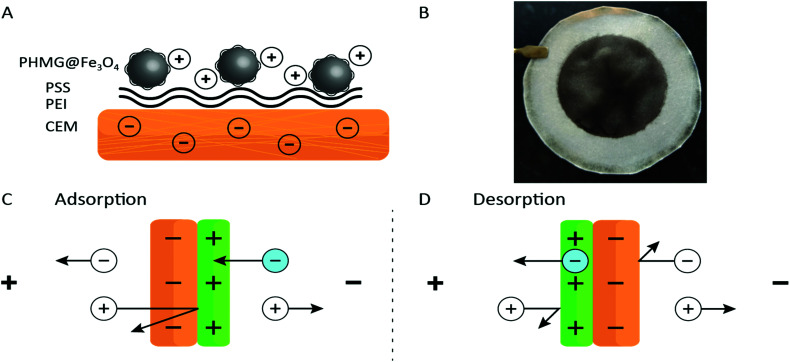
(A) Schematic illustration of the membrane composed from a CEM support, two intermediate (cushion) polyelectrolyte layers and PHMG-coated Fe_3_O_4_ NPs. (B) Photograph of the membrane modified with the PHMG@Fe_3_O_4_ (diameter of the black coating equals to 47 mm). (C) Schematic of the adsorption and (D) desorption processes in the ED setup. The schemes are not to scale; the PHMG@Fe_3_O_4_ coating is much thinner than the CEM.

After immobilizing all chemical building blocks to the CEM surface, a clear and relatively homogenous black coating was obtained – see Fig. 1B (further information about the coating process can be found in Section S5 of the ESI†). The formed deposit proved to be stable as no delamination was observed upon thorough rinsing or even after a few hours flushing with the working solutions in the ED set-up.

The working principle of the adsorptive ion-exchange membrane is presented in Fig. 1C and D. During adsorption, a positive current is applied to the ED set-up with the PHMG@Fe_3_O_4_ NPs membrane site facing towards the cathode. As such, negatively charged ions (in this case phosphate) from the feed solution compartment can only penetrate the positively charged coating and will be rejected at the interface with the CEM. At the same time, cations from the receiving compartment will go through the CEM and should be rejected (assuming 100% coating efficiency, which is not the case, *vide infra*) by the positively charged coating. During the desorption step, the membrane can be inverted (the coated side facing towards the anode) or the polarity of the counter electrodes can be changed. As explained above, using electrochemistry, phosphate can be released at the same time regenerating the adsorptive coating. Due to charge–charge repulsive effects, anions and cations from the feed and receiving compartments, respectively, will be rejected (again assuming 100% efficiency) by the membrane.

Batch adsorption experiments were performed to estimate the phosphate adsorption capacity. Net positively charged coacervates were fabricated by the addition of PHMG to PSS solution in a molar ratio 2 : 1 (pertaining to the charged functionalities of both polyelectrolytes), giving a solution as shown in Fig. 2A (left picture). Particle sizes in the micrometer range were found, based on DLS measurements, with a zeta potential equal to +37 ± 5 mV (measured at pH = 7). Experimental details and the full characterization of the obtained coacervates can be found in Sections S2 and S3 of the ESI.† The formed suspension of particles was easy to separate by centrifugation. A white gel-like precipitate was formed and the composition of the supernatant was further analyzed by ion chromatography (IC). Fig. 2B shows the results of the phosphate adsorption to the coacervates. During experiments the initial phosphate concentration was varied from 1.6 mM to 50.5 mM, while keeping the ratio of PHMG : PSS : PO_4_^3−^ equal to 2 : 1 : 1. It was found that *ca.* 11% of the phosphate adsorbs onto the exposed guanidinium groups. The apparently increasing absorption capacity for higher PO_4_^3−^ concentrations probably originates from different morphology and size of the formed coacervates. This experiment proved that PHMG is capable of adsorbing phosphate.

**Fig. 2:**
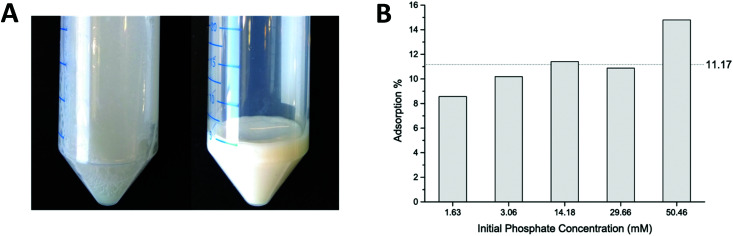
(A) Coacervates solutions before (right) and after (left) centrifugation ([PHMG] : [PSS] : [PO_4_^3−^] = 2 : 1 : 1). (B) The result of a coacervates batch adsorption experiment. The ratio of PHMG : PSS :  PO_4_^3−^ was constant and equal to 2 : 1 : 1.

With Scanning Electron Microscopy – Energy Dispersive X-ray Spectroscopy (SEM-EDS) the coating stability was examined. The results are given in Section S5 and Fig. S3 of the ESI.† It was found that the PHMG@NPs remain at the membrane surface even after thorough rinsing with water, which stresses its practical utility.

The performance of a PHMG@NPs@CEM was assessed in an ED set-up (Fig. S4 together with the experimental methodology described in Section S6 of the ESI†). Briefly, the membrane under investigation was placed in a holder positioned between the feed and receiving compartments of the ED setup, which contain a 0.2 M NaH_2_PO_4_ and a 0.2 M NaCl solution, respectively. During the adsorption step, the coated side was always placed facing towards the feed compartment and a current of 100 mA was applied for 90 seconds. Next, the membrane was inverted and the same current was applied for 5 min (desorption step). This cycle was repeated 5 times. After each step, a 10 mL sample was taken from the receiving compartment to determine the concentration of released PO_4_^3−^ using IC (Section S7 of the ESI†). In both steps, a gradual increase in [PO_4_^3−^] in the receiving compartment was found and the results are shown in Fig. 3A (red bars). Simultaneously, we performed two blank experiments (i) with the bare CEM placed in the ED holder without any electric current applied (Fig. S5, ESI†) and (ii) with the electric current applied between the two counter electrodes (Fig. 3A (white bars)). As the observed [PO_4_^3−^] in the receiving compartment increased at almost the same rate for two blank experiments, we concluded that this increase originates from the phosphate species physically adsorbed to the membrane and its holder and that they desorbed after membrane inversion to the receiving compartment (present although the membrane was rinsed with distilled water between each step). This increase is depicted in Fig. 3A and Fig. S5 (ESI†), was always present and it was consistent and reproducible. Therefore, the phosphate release for the bare CEM was used as a baseline for the adsorption experiments with PHMG@NPs@CEM. Fig. 3A (red bars), shows the increase in [PO_4_^3−^] in the receiving compartment of an ED cell when the PHMG@NPs@CEM was used. The graph shown in Fig. 3B was obtained by subtracting the amount of phosphate transported into the receiving compartment by the CEM from the one obtained using PHMG@NPs@CEM. This way, the phosphate transported during the desorption step from the PHMG@NP-based coating into the receiving compartment can be probed and evaluated. Analysis of Fig. 3B reveals that the [PO_4_^3−^] increment released to the receiving compartment after each desorption cycle is 27 ± 13 mM. During 5 cycles, we have recovered *ca.* 0.055 mmol phosphate, which equals to *ca.* 0.21 mg P cm^−2^ for a used membrane area of 8.14 cm^2^. While both the membrane coating process and the adsorption process still require optimization, this result nonetheless proves that the concept of combining adsorption with an ED process for ion recovery is very promising and should be further investigated. We aim to develop this system by (i) optimizing the amount of adsorbent placed at the ion-exchange membrane surface; (ii) incorporating different functional groups into the coating and/or (iii) understanding the role of competing ions transport across the proposed type of a membrane.

**Fig. 3:**
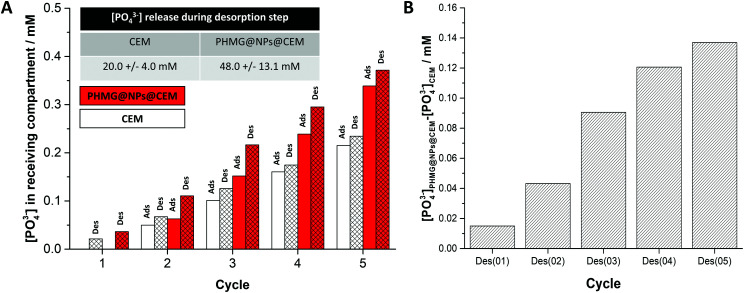
(A) [PO_4_^3−^] in the receiving compartment of the electrodialysis cell measured during adsorption/desorption experiments. The numbers on the horizontal axis correspond to each cycle, and for each experiment, the [PO_4_^3−^] in the receiving compartment is shown after the adsorption and the desorption step. Normalization was performed to the first measured phosphate concentration (during run 0). (B) Amount of phosphate transported into the receiving compartment of the electrodialysis cell by the anionic coating – obtained by subtracting the amount of phosphate transported into the receiving compartment by the CEM from the one obtained using PHMG@NPs@CEM.

This study offers a new concept for the ion separation for water treatment applications. It successfully combines an adsorption process with an electrodialysis system. In the future, we plan to improve our system *via* modification and optimization of the coating layer, followed by a thorough characterization study involving all ions undergoing transmembrane transfer or rejection. Different ions will be targeted with the help of synthetic chemistry and incorporation of other ion-specific chemical functionalities into NP-based coatings.

This work is part of a research program titled ‘Modular Functionalized Ceramic Nanofiltration Membranes’ (BL-20-10), which is taking place within the framework of the Institute for Sustainable Process Technology (ISPT, The Netherlands) and is jointly financed by the Netherlands Organization for Scientific Research (NWO, The Netherlands) and ISPT. LCPMdS acknowledges the European Research Council (ERC) for a consolidator Grant, which is part of the European Union's Horizon 2020 Research and Innovation Program (grant agreement No. 682444). Laura P. is grateful to Wetsus (NL) for financial support.

## Conflicts of interest

There are no conflicts to declare.

## Supplementary Material

CC-056-C9CC09563B-s001
