# Supplementary material for: Modified cation-exchange membrane for phosphate recovery in an electrochemically assisted adsorption–desorption process
Source: Chem Commun (Camb). 2020 Mar 27;56(37):5046–9. doi: 10.1039/c9cc09563b (PMC8610146; doi:10.1039/c9cc09563b)
Supplement: CC-056-C9CC09563B-s001 [file CC-056-C9CC09563B-s001.pdf]

Electronic Supplementary Material (ESI) for Chemical Communications.
This journal is © The Royal Society of Chemistry 2020

Supporting Information to

Modified Cation-Exchange Membrane for Phosphate Recovery in Electrochemically Assisted Adsorption-Desorption Process

Kostadin V. Petrov,[a] Laura Paltrinieri,[a,b] Lukasz Poltorak,[a,c]* Louis C.P.M. de Smet,[a,d] and Ernst J. R. Sudhölter[a]

a. Delft University of Technology, Department of Chemical Engineering, Van der Maasweg 9, 2629 HZ Delft, The Netherlands.
b. Wetsus – European centre of excellence for sustainable water technology, Oostergoweg 9, Leeuwarden 8932 PG, the Netherlands
c. Department of Inorganic and Analytical Chemistry, Faculty of Chemistry, University of Lodz, Tamka 12, 91-403 Lodz, Poland
d. Laboratory of Organic Chemistry, Wageningen University & Research, Wageningen, The Netherlands.

*lukasz.poltorak@chemia.uni.lodz.pl

Supporting Information Outline

S1	Chemicals	page 2
S2	Coacervates – protocol of preparation and characterization	page 2
S3	Protocol – phosphate batch adsorption experiments using coacervates	page 3
S4	Fe_3O_4 NPs modification and batch adsorption experiment	page 4
S4.1	TGA Analysis	page 4
S4.2	FTIR measurement	page 5
S4.3	Zeta-potential measurements of the PHMG@Fe_3O_4 NPs	page 6
S5	Membrane modification	page 7
S5.1	Membrane characterization	page 7
S6	Adsorption experiments in the electrodialysis cell	page 9
S7	Ion chromatography analysis	page 12
S8	References	page 12

S1 Chemicals

PHMG (≥ 99% purity) was supplied by Sinotech (China) and was used without any further purification. The Cation-Exchange Membranes (CEMs) used in this work were cut from a 50cm × 2m roll of type 2 CEM produced by Fujifilm Manufactory Europe. The ion-exchanging sites of the CEM are sulfonate groups. Poly(sodium 4-styrenesulfonate) (PSS, M.W. ≈ 70,000), branched polyethyleneimine (PEI, M.W. ≈ 25,000), sodium phosphate monobasic monohydrate ($NaH_2PO_4 \cdot H_2O$), ≥ 99:5%, chloride standard for IC (1000 ± 4 mg/L), and phosphate phosphorus standard for IC (1000 ± 4 mg/L), sodium chloride (NaCl, ≥ 99%) and iron oxide nanoparticles in the form of Iron(II,III) oxide nanopowder with a 50-100 nm diameter were all purchased from Sigma-Aldrich. Hydrochloric acid solution (HCl, 1 M), sodium hydroxide solution (NaOH, 1 M), sulphuric acid (H_2SO_4 95-97 %) were bought from Merck. Milli-Q water was purified using a Milli-Q IQ-7000 unit.

S2 Coacervates – protocol of preparation and characterization

To study the optimal molar ratio between PSS and PHMG, five 50 mL solutions were created. Table S1 shows the molar quantities and the molar ratio between the two polyelectrolytes. It is important to note that the PSS solution was added dropwise (over two min.) to the PHMG solution, and not the other way around. This was to ensure that PHMG's positively charged groups were on the outside of the coacervates particles.

Table S1. Overview of the composition of the PHMG/PSS solutions.

Solution Number	n(PHMG), (mol)	n(PSS), (mol)	Molar ratio (PHMG/PSS)
**1**	2.83×10^{-4}	2.83×10^{-4}	1:1
**2**	2.83×10^{-4}	1.41×10^{-4}	2:1
**3**	2.83×10^{-4}	2.81×10^{-5}	10:1
**4**	2.83×10^{-4}	1.34×10^{-5}	21:1
**5**	2.83×10^{-4}	5.65×10^{-4}	1:2

The size distribution and the zeta potential of the formed particles were analyzed, using dynamic light scattering (DLS) technique with a Malvern Zeta-sizer instrument. Table S2 displays the results of this analysis. The coacervates were positively charged for the PHMG:PSS function group molar ratio equal to 1:1 or for the solutions where the number of guanidinium groups was used in excess. As expected, when the PSS used was in excess, charge reversal at the surface of the coacervates occurred. The solution with a PHMG:PSS equal to 2:1 was chosen for further experiments. This was because the formed coacervates formed visible agglomerates (see Fig. 2A in the main text) that were easily to separate by centrifugation.

Table S2. Zeta sizer results from coacervate solutions analysis, for optimal ratio.

Ratio (PHMG:PSS)	ζ-potential (mV)	Size (nm)
**1:1**	11.5 ± 3.0	4041*
**2:1**	37.4 ± 5.0	5267*
**10:1**	46.0 ± 6.4	59
**21:1**	27.8 ± 5.6	40
**1:2**	-49.2 ± 8.5	3739*

* Low-quality data fit.

The fluctuation in the coacervate size (see Table S2) can be controlled by the careful balance of the charge ratio between functional groups located within polymer structure. When one of the polyelectrolytes is used in excess, the formed particles surface charge is rapidly saturated and their further growth is inhibited by electrostatic repulsion. In turn, particles continue to grow and agglomeration can be triggered close to the isoelectric point.[1]

S3 Protocol – phosphate batch adsorption experiments using coacervates

In order to prove the ability of coacervates to adsorb phosphate, five solutions (50 mL each) containing PHMG:PSS at a ratio equal to 2:1 (concentration of PSS was varied from 1.5 mM to 50 mM) were prepared. The coacervate solutions were stirred for 24 h. Next, the phosphate was added to each solution so that the final molar ratio was 2:1:1 (PHMG:PSS:PO_4^{3-}). The obtained solutions were stirred for 24 h followed by centrifugation at 5000 rpm for 5 min using a Hermle Z 326 K centrifuge. Finally, 10 mL of a supernatant was taken and analyzed by ion chromatography (IC). The results of this analysis can be found in Figure 2B available in the main text of this study.

S4 Fe_3O_4 NPs modification and batch adsorption experiment

Two separate aqueous solutions of PHMG (2.5 g/L) and Fe_3O_4 NP suspension (0.5 g/L) were prepared using a ultrasonication bath for 20 min. Next, the pH of both solutions was adjusted to 9.5 by the addition of concentrated HCl or NaOH (1 M). At this pH the surface of Fe_3O_4 NPs and the polyelectrolyte is expected to be negatively and positively charged, respectively. Next, the Fe_3O_4 NP suspension was added drop-wise to the PHMG solution and stirred for 24 h at room temperature to ensure the complete adsorption of PHMG to the Fe_3O_4 NP surface. Afterward, the NPs were collected with a magnet and the supernatant solution containing the excess of the polymer was removed. The NPs were re-dispersed in Milli-Q water. This procedure was repeated 3 times. The resulting coated Fe_3O_4 NPs (PHMG@Fe_3O_4) were analyzed using thermogravimetric analysis (TGA), Fourier transform infrared spectroscopy (FTIR) and ZetaSizer (Malvern) allowing for the particle size distribution, functional group analysis and ζ-potential determinations. Bare Fe_3O_4 NPs were also analyzed and used as a blank.

S4.1 TGA Analysis

The bare and modified NPs were dried in a Binder oven at 50 °C for 24 h. The TGA analysis was performed in a GA2/SF1100 STARe system from Mettler Toledo. Between 7 and 10 mg of dry NP were exposed to a temperature increase from room temperature up to 800 °C, at a rate of 5°C per min. The analysis was performed in a nitrogen instead of an air environment as the iron (II), present in the magnetite particles, will be oxidized to iron (III). Working under nitrogen also means that the obtained result can be only treated qualitatively (we follow calcination rather than oxidation). The observed weight loss of the organic part informs about the degradation (carbonization) only and not the complete oxidation of the polymer. Figure S1 displays the TGA of modified and unmodified NPs. The presence of the PHMG coating was confirmed by analyzing the differences in weight loss between the two measurements. While the bare NPs are stable throughout the measurement, the modified NPs show a weight loss that we attributed to the polymer degradation. The weight loss of only 3% is not surprising as the PHMG forms a thin layer on the magnetite NPs surface.

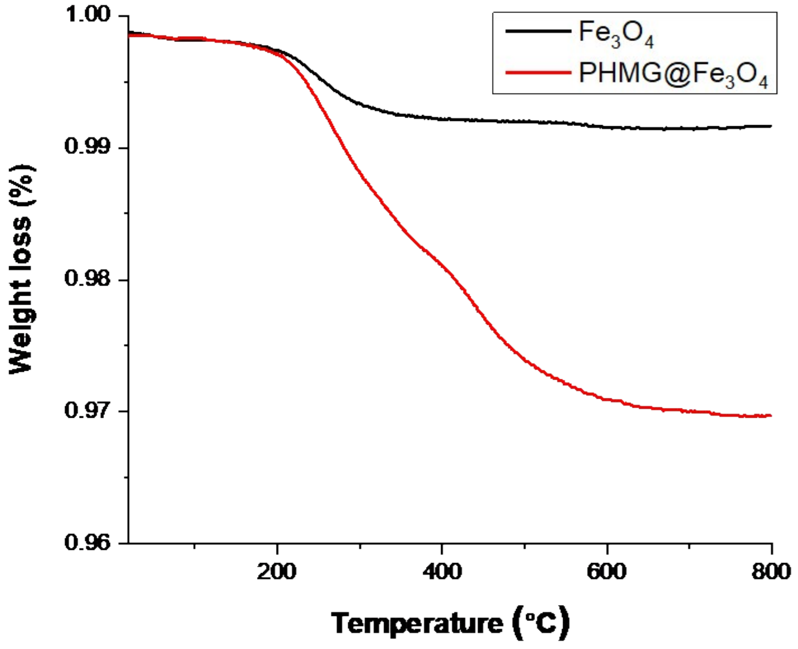

Figure S1. *TGA result of Fe_3O_4 NP and PHMG@Fe_3O_4 NP in N_2.*

S4.2 FTIR analysis

For the FTIR measurements, the NPs were removed from the solution and dried in the way as described for the TGA experiments. Next, a KBr pellet was made using a manual press. Fig. S2 shows the two relevant regions between 4000 and 2700 cm^{-1} and 1000 and 500 cm^{-1}, that correspond to the peaks of interest. In more detail, the broad peak with a center at 3437 cm^{-1} is associated with the O-H bond stretching, originating from the H_2O molecules entrapped within the polyelectrolyte chain. Two peaks that indicate the presence of the polymer can be observed at 2925 and 2857 cm^{-1} and they are associated with the asymmetric and symmetric stretching of C-H bonds, respectively[2,3]. The intensity of these peaks could also signify that the amount of PHMG at the surface of NPs is rather low. The peak at 596 cm^{-1} confirms the presence of iron oxide as it is associated with the Fe-O bond[4]. The noise recorded in the spectral range between 4000 cm^{-1} and 3500 cm^{-1} is due to the water vapor present in the measurement chamber.[5]

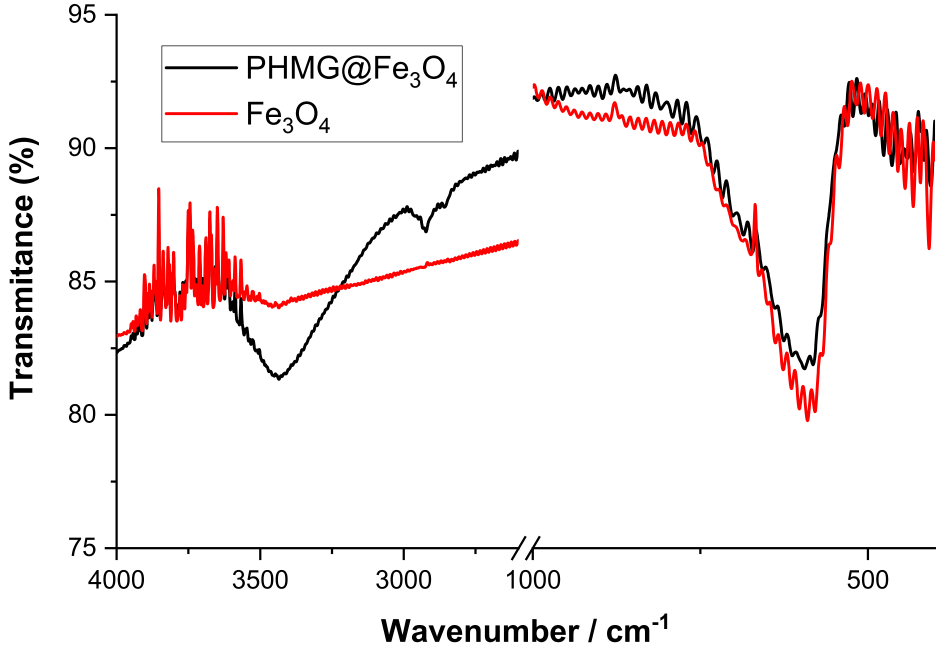

Figure S2. FTIR region spectra of coated and bare Fe_3O_4 NPs.

S4.3 ζ-potential measurements of the PHMG@Fe_3O_4 NPs

The ζ-potential analysis was performed with a Malvern Zeta-Sizer. The dry NPs powder was dispersed in Milli-Q water and sonicated for 10 min. The results are summarized in Table S3. The ζ-potential after the modification is significantly higher as compared with the bare NPs.

Table S3. ζ-potential analysis of Fe_3O_4 NPs and PHMG@Fe_3O_4 NPs (pH = 7).

Sample	ζ-potential (mV)
**Fe_3O_4 NPs**	6.23 ± 5.7
	3.4 ± 5.1
**PHMG@Fe_3O_4 NPs**	48.6 ± 3.6
	50.5 ± 3.6
	51.9 ± 4.2

S5 Membrane modification

The CEM was initially activated by argon plasma treatment (2 min) in a Harrick Plasma PDC-002-CE plasma cleaning equipment, at a high RF level. This step had the aim of generating free radicals, which could potentially react with the CEM surface and increase its charge. [6] Afterward, the membrane was placed in a Millipore filtration holder, simply to provide support for the coating process. Next, 2 mL (enough to cover the membrane surface) of a branched polyethyleneimine (PEI) solution with a concentration of 1 g/L was casted on top of the membrane. It was kept there for 5 min followed by thoroughly rinsing with MilliQ water. Next, 2 mL of a PSS (1 g/L) solution was placed at the membrane surface and kept for 5 min followed by a washing step. Finally, 3 mL of a 0.5 g/L solution of PHMG@Fe_3O_4 NP was added and left for 24h, giving 8.6 mg/cm^2 of the deposit at the membrane surface

S5.1 Membrane characterization

After the modification, the presence and stability of the coating were confirmed by scanning electron microscopy (SEM) and energy-dispersive X-ray spectroscopy (EDS) analysis before and after the membrane had been kept in demi-water for 1 h followed by thoroughly rinsing under a constant stream of MilliQ water. The SEM and EDS results before and after rinsing step are shown in Fig. S3. SEM micrographics are given on top of Fig. S3A and S3B, where the lighter areas correspond to the coated Fe_3O_4 NPs, and the darker areas correspond to the CEM material. The elongated features that can be observed on the carbon, oxygen and sulfur EDS results (see bottom of Fig. 3A and 3B), are the polypropylene (PP) fibers which are in the constitution of the Fuji CEM to enhance its mechanical properties. The carbon EDS results show this element abundancy (the area with most intense signal corresponds to the PP fibers). Again, for the carbon EDS mapping, the less intense areas show a pattern, which is related to the fact that the PHMG@Fe_3O_4 NPs cover part of the membrane. The same pattern can be found on the iron, oxygen and sulfur EDX mapping images. The existence of areas with iron and oxygen showing a more intense signal (as compared with carbon and sulfur) confirms the presence of Fe_3O_4 NPs. Fig. 3B shows similar set of micrographics after thorough membrane rinsing. It can be observed that the membrane exhibits very similar morphology. The NPs count remains the same (see on the SEM images in Fig. 3A and 3B), and the iron and oxygen do not diminish on the corresponding EDS mapping micrographics and the images also look comparable. All combined results confirm the superior stability of the coating.

Furthermore, electrical resistance (ER) was measured in the electrodialysis setup which is described in section S6. A potentiometric measurement was used, by applying a current from 0 to ≈ 0.3 A and

measuring the potential difference across the membrane. The measurement was performed in a solution of 0.5 M sodium phosphate (NaH_2PO_4) (pH=5). As it can be observed in Table S4, the ER is reduced in the presence of the coating. A possible explanation for this is that the interface between the anionic coating and the CEM, resembling a bipolar membrane, is responsible for a higher water dissociation rate at a bipolar junction in the presence of externally applied current. This, in turn, can result in the formation of H^+ or OH^- ions which have higher mobility, and by carrying the current which is being applied, they reduce the resistance. Although this is likely the case, it is just a speculation as the pH at the surface of the membrane is very challenging to be measured.

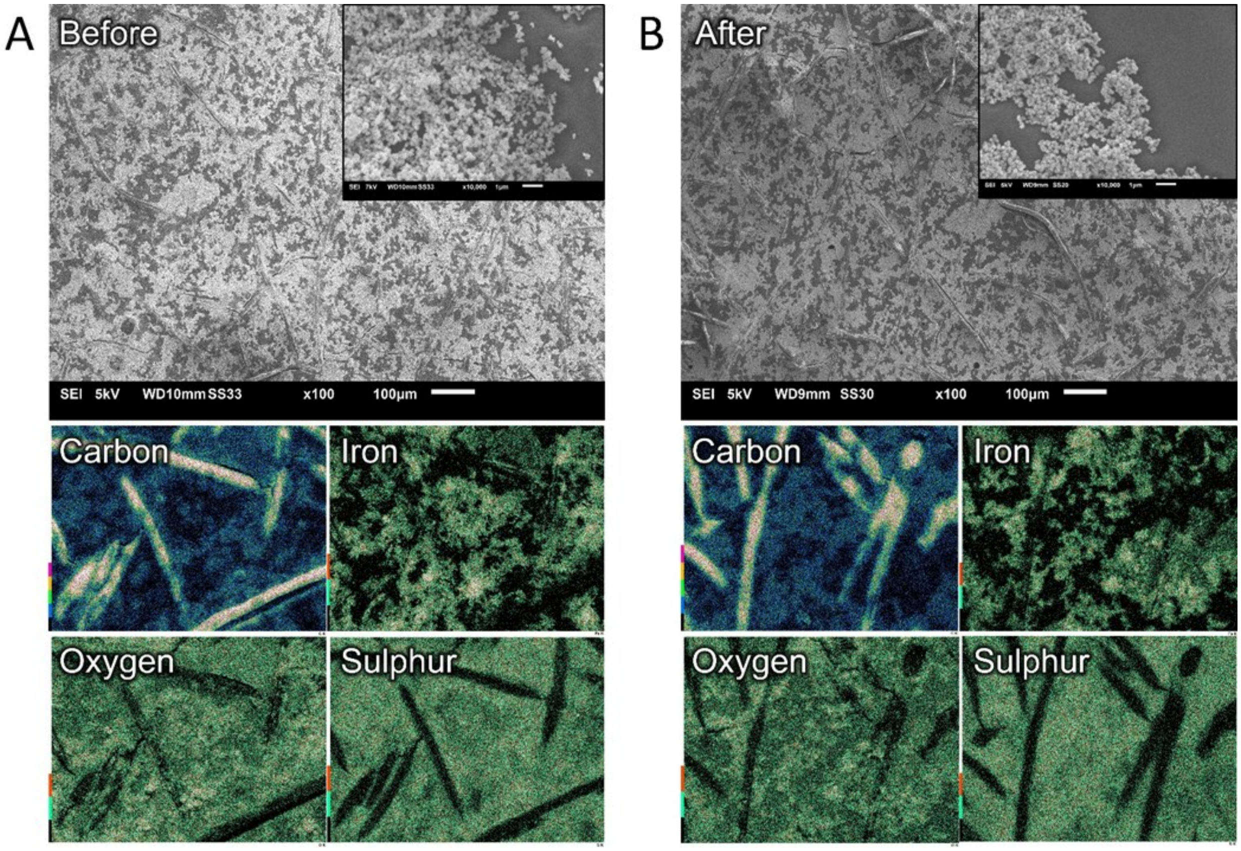

Figure S3. SEM-EDS analysis of the PHMG@NPs@CEM before (A) and after (B) rinsing with water. The SEM and EDS micrographs were taken at an 100× and 500× amplification, respectively.

Table S4. Electrical resistance analysis of PHMG@NPs@CEM in 0.5M NaH_2PO_4 (pH=5).

Sample	Electrical Resistance (Ω)
**CEM**	6.56 ± 0.08
**PHMG@NPs@CEM**	5.97 ± 0.09

S6 Adsorption experiments in the electrodialysis cell.

Fig. S4 shows a schematic illustration of the electrodialysis (ED) setup used for the adsorption-desorption experiments. The prepared membrane was placed in ultrapure Milli-Q water for 1 h before the experiments. After this time it was placed into the setup (position between compartment 3 and 4), with the PHMG@Fe_3O_4 coated side facing towards the cathode. The feeding compartment (4) was filled with a 0.2M NaH_2PO_4 solution (pH=5), and the receiving compartment (3) with a 0.2M NaCl solution. Both solutions were in constant flow triggered by a MasterFlex L/S pump operating at 327 rpm. During the phosphate adsorption step, a positive current of 100mA was applied for 90 seconds. After this time the sample from compartment 3 was collected for IC characterization. Next, the setup was emptied and the membrane was dipped in distilled water, in order to remove any remaining phosphate adhering to the membrane and the sample holder surface. Before the desorption step, the membrane was placed in the ED setup with the coating facing the anode. All compartments were filled and the positive current (100 mA) was applied for 300 sec to promote desorption. During each experiment, five adsorption-desorption cycles were performed. A 10 mL aliquots were probed from the solution leaving the receiving compartment before the experiment was started, after each adsorption and desorption step. The concentration of phosphate in this solution was followed by IC.

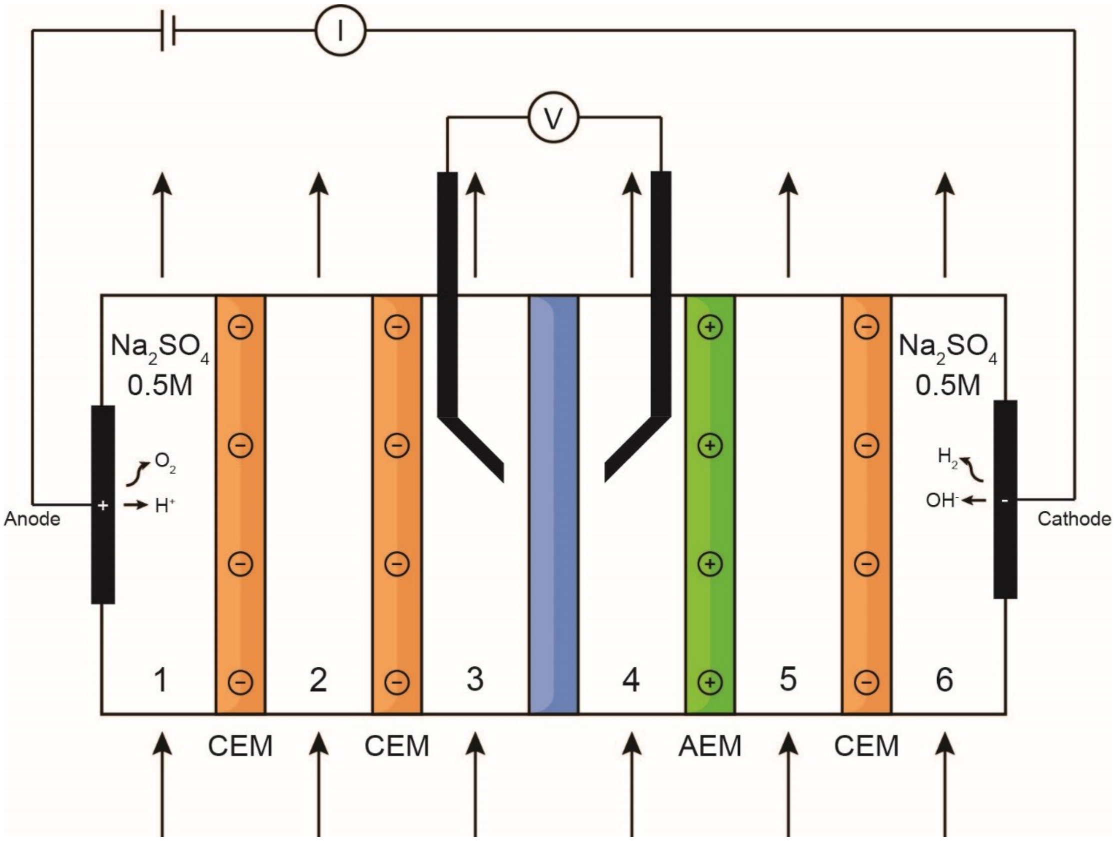

Figure S4. Schematic Illustration of the ED setup. The investigated membrane (in our case, PHMG@NPs@CEM) was placed in a sample holder with an opening having 8.14 cm^2 situated between compartments 3 and 4, these being the receiving and the feed compartment respectively. Compartments 2 and 3 contained 0.2 M solution of NaCl whereas compartments 4 and 5 contained 0.2 M solution of NaH_2PO_4.

For the blank experiments, we repeated the protocol described above for the unmodified CEM in the (i) presence and (ii) absence of the applied electric current. For both scenarios, we observed a gradual and similar increase in phosphate concentration in the receiving compartment. This, in turn, suggest that it is physical adsorption of phosphate to the membrane support rather than transmembrane transport. Also, the phosphate concentration in the receiving compartment found during the blank experiment was always lower than that for the modified membrane.

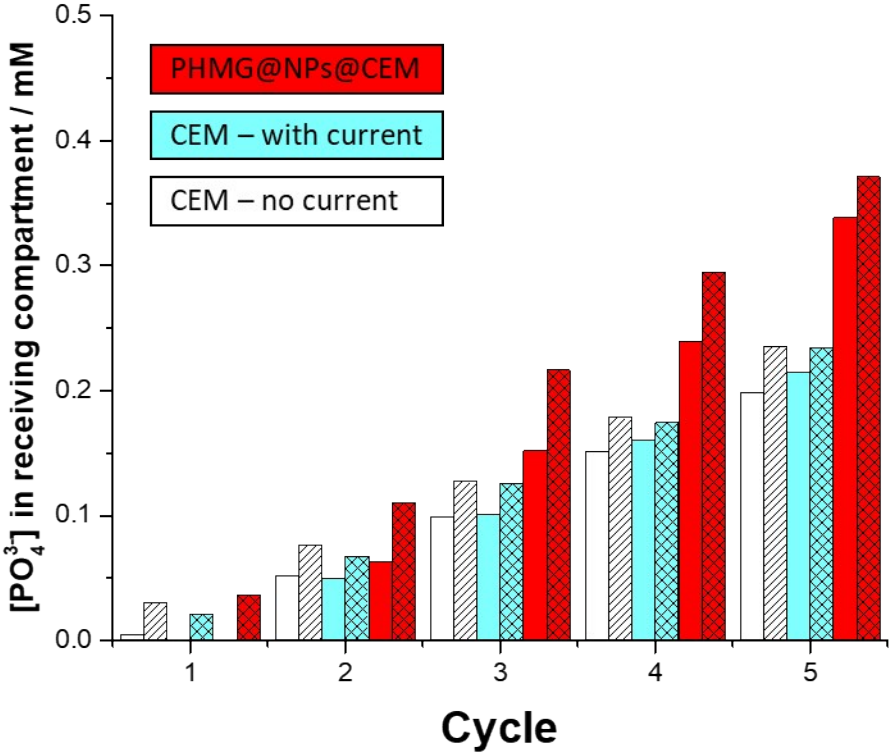

Figure S5. [PO_4^{3-}] in the receiving compartment of the electrodialysis cell measured during the adsorption/desorption experiment. Red bars – the membrane modified with the PHMG@NPs; Blue bars – the blank experiment with CEM and applied electric current; White bars – the blank experiment with CEM in the absence of the electric current. Clean and pattern bars correspond to the adsorption and desorption step respectively.

S7 Ion chromatography analysis

All samples from adsorption/desorption experiments and batch adsorption experiments were filtered using a 0.45 µm Supor® PES filter and analyzed in an 881 Compact IC Pro, 150 mm A Supp 5 column, Metrohm equipment. A mixture of sodium carbonate and sodium bicarbonate (339 mg/L and 84 mg/L, respectively) was used as the eluent, and a 0.3M solution of H_3PO_4 was used as a regenerator. The samples produced in the coacervate-based experiment were additionally filtered using the 0.2 µm PVDF filter.

S8 Reference

1 F. Quemeneur, M. Rinaudo, G. Maret and B. Pépin-Donat, *Soft Matter*, 2010, **6**, 4471–4481.

2 M. J. Baker, C. S. Hughes and K. A. Hollywood, *Biophotonics Vib. Spectrosc. Diagnostics*, 2016, 2–14.

3 M. J. Bertrand, *J. Am. Chem. Soc.*, 1998, **120**, 6633.

4 T. Wang, L. Zhang, H. Wang, W. Yang, Y. Fu, W. Zhou, W. Yu, K. Xiang, Z. Su, S. Dai and L. Chai, *ACS Appl. Mater. Interfaces*, 2013, **5**, 12449–12459.

5 Shimadzu. Excellence in Scinece., https://www.shimadzu.com/an/ftir/support/faq/4.html, (accessed 6 March 2020).

6 L. Paltrinieri, E. Huerta, T. Puts, W. van Baak, A. B. Verver, E. J. R. Sudhölter, L. C. P. M. de Smet, *Enivornmental Sci. Technol.* 2018, **53**, 2396 - 2404
